# QSAR Studies on *N*-aryl Derivative Activity Towards Alzheimer’s Disease

**DOI:** 10.3390/molecules14041448

**Published:** 2009-04-07

**Authors:** Kamalakaran Anand Solomon, Srinivasan Sundararajan, Veluchamy Abirami

**Affiliations:** Department of Bioinformatics, Sri Ramachandra University, No.1.Ramachandra Nagar, Porur, Chennai- 600116, India

**Keywords:** *N*-aryl derivatives, Alzheimer’s disease, Genetic Function Approximation, QSAR

## Abstract

A Quantitative Structure Activity Relationship (QSAR) study has been an attempted on a series of 88 *N*-aryl derivatives which display varied inhibitory activity towards both acetylcholinesterase (AChE) and butyrylcholinesterase (BChE), targets in Alzheimer’s drug discovery. QSAR models were derived for 53 and 61 compounds for each target, respectively, with the aid of genetic function approximation (GFA) technique using topological, molecular shape, electronic and structural descriptors. The predictive ability of the QSAR model was evaluated using a test set of 26 compounds for AChE (r^2 ^_pred _= 0.857), (q^2 ^= 0.803) and 20 compounds for BChE (r^2 ^_pred _= 0.882), (q^2 ^= 0.857). The QSAR models point out that AlogP98, Wiener, Kappa-1-AM, Dipole-Mag, and CHI-1 are the important descriptors effectively describing the bioactivity of the compounds.

## 1. Introduction

Alzheimer’s disease (AD), the most common form of dementia among the aged, is a fatal neurodegenerative disease characterized by loss of mental ability, cognition deterioration, progressive impairment of daily activities and a variety of neuropsychiatric symptoms and behavioral disturbances [1,2]. Both acetylcholinesterase and butyrylcholinesterase are enzymes whose vital function is the hydrolytic breakdown and degradation of acetylcholine (ACh), a neurotransmitter which plays a role in the modulation of memory function in normal and neurodegenerative conditions [3]. Choline esterase is the only target that has resulted in the design of a few palliative drugs presently marketed for the treatment of the Alzheimer’s disease [4]. Tacrine, galantamine, rivastigmine, donopezil and huperzine are some cholinesterase inhibitors, currently used promising drugs for the treatment of Alzheimer’s disease [5]. But their clinical use is strictly limited because of several adverse effects such as hepatotoxicity and some pharmacokinetic disadvantages, so the study of new compounds as cholinesterase inhibitors is required to discover more effective and targeted drugs. 

The Quantitative Structure Activity Relationship (QSAR) methodology is useful in predicting the activity of novel molecules by mathematical equations which deduce the relationship(s) between a chemical structure and its biological activity [6,7,8,9,10,11,12]. QSAR models are pointers to design effective drugs. QSAR studies on Alzheimer’s disease targets has been carried out on phenylpentenone derivatives [13], physotigmine analogues [14], indanone [15], and tacrine [16]. 

Recent studies indicate that *N*-aryl derivatives (amides and imides) to be active-site-directed inhibitors of acetylcholine esterase and butyrylcholine esterase [17]. For the present study, a QSAR study for a series of 88 *N*-aryl compounds (whose inhibitory effect against Ache and Bche was reported in the above paper) was carried out using the Cerius2 package. The QSAR module in this package provides different descriptors that are categorized into different types like spatial, structural, electronic, conformational, thermodynamic and receptor. A QSAR model was generated using the Genetic Function Approximation (GFA), which has also been applied for the QSAR analysis of steroids, dopamine β-hydroxylase inhibitors [18] and anticancer agents [19]. An interesting application of GFA is in the QSAR studies on acetylcholinesterase inhibitors which has already resulted in discovery of a new molecule, E2020, for the treatment of Alzheimer’s disease [20]. 

## 2. Results and Discussion

Out of 88 *N*-aryl derivatives, the QSAR models were generated using 53 and 59 compounds as training set for acetylcholinesterase and butyrylcholinesterase, respectively. Significant r^2^ values of 0.862 and 0.887 were obtained using the GFA technique. The best QSAR model, chosen based on the statistical values for acetylcholinesterase (equation 1) and butyrylcholinesterase (equation 2) are given below:

Equation 1: pK_i_ = 3.05473 + 0.005491 * (WIENER) + 0.228406 * (ALOGP98) -4.18285 * (CHI-V-3_C) -0.546045 * (PHI) r^2 ^= 0.862, r^2 ^_adj _= 0.856, LOF = 0.021, q^2 ^= 0.803.

Equation 2: pKi = 3.79965 -0.604278 * (KAPPA-1-AM)+ 0.005604 * (WIENER)+ 0.333816 * (CHI-1) + 0.039435*(DIPOLE-MAG) + 0.251838*(ALOGP98) r^2 ^=0.884, r^2 ^ adj =0.873, LOF=0.032, q^2 ^= 0.857.

The predictive power of the QSAR equations was assessed using a test set of 26 compounds for acetylcholinesterase and 20 compounds for butyrylcholinesterase inhibitors with regularly distributed biological activities similar to those in the training sets. The structural features, biological activity and molecular descriptors of all the compounds used in the training set for both Ache and Bche are given in the Supplementary Material. 

The physical/ chemical / physiochemical significance of each of the descriptors appearing in the QSAR equations are given in Table 1.

**Table 1:** Significance of descriptors used in this QSAR study.

Descriptor	Type	Significance
ALOGP98	Thermodynamic descriptor	Logarithm of partition coefficient
WIENER	Graph–theoretical descriptor	Sum of chemical bonds between atoms
CHI-V-1-3	Topological descriptor	Molecular connectivity indices
CHI-1		
KAPPA-1-AM	Topological descriptor	Molecular Shape Kappa indices
DIPOLE-MAG	Electronic descriptor	Dipole moment
PHI	Topological descriptor	Molecular flexibility indices
LogZ	Topological descriptor	Logarithm of Hosoya index
HBOND DONOR	Structural descriptor	Number of hydrogen bond donors groups

Out of the total 88 *N*-aryl compounds, 79 were selected for QSAR analysis in Cerius2 using GFA technique, the remaining being left out due to poor scalability. Genetic Function Approximation (GFA), statistical analysis was carried out for all 79 compounds for acetylcholine esterase and butyrylcholine esterase respectively. 

To improve the predictive power of the QSAR model, outliers were removed from the sets. In the case of the Ache inhibitors, the log ki values of 26 compounds in the test set (out of 88) were completely out of the scalable range of the remaining 60 compounds. Hence, for better scalability, they were treated as outliers and not included in the training set for QSAR calculations. Likewise 20 Bche inhibitors were ignored for the same reason. Using Leave-one-out (LOO) method, final training sets of 53 and 61 compounds were selected and the best equations were obtained with the combination of 2D topological, thermodynamic, structural, electronic, charge dependent descriptors.

Out of the 43 descriptors calculated for each compound in the dataset, only a few, *viz*., CHI-V-1-3, CHI-1, WIENER, ALOGP98, KAPPA-1-AM , DIPOLE-MAG, PHI, LogZ and HBOND DONOR were selected based on the correlation coefficient r (0.929, 0.942), squared correlation coefficient (0.862, 0.887) and cross validated r^2^ or q ^2^ (0.803, 0.857) of the model generated. Recent QSAR studies on AchE inhibitors [21] have highlighted log P, NORB and WNSA1 as deterministic descriptors in favor for the AChE activity using GFA technique. This too was observed in this study, where ALOGP98 and WIENER appear in the QSAR equation in both the cases *viz*., AChE and BChE. Therefore, all the above mentioned descriptors can be used as filters while exploring a database or as an important criterion while designing new chemical entities as leads for effective drugs to function as AChE/BChE inhibitors.

To validate the final QSAR model, test sets of 26 and 20 compounds were used for acetylcholinesterase and butyrylcholinesterase inhibition, respectively. The predictive power of the model was reasonably good with predictive r^2^, (r^2 ^_pred_) value of 0.857 and 0.882, cross validated r^2 ^(0.929, 0.917), respectively, for the set of compounds against Ache and Bche. Structure and statistics of the training set is given in the supplementary data. The experimental and predicted values of the biological activity are given in Table 2 and Table 3.

**Table 2:** Experimental and predicted biological activity of test set of compounds against AChE. 
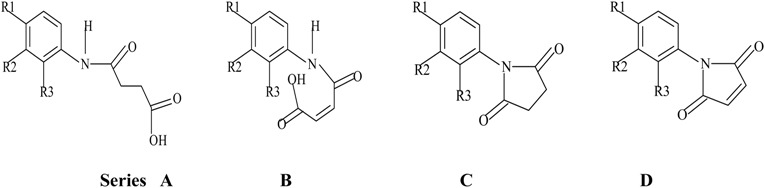

Compd. No.	Substituents			Experimental Activity	GFA Predicted Activity	GFA Residuals Activity
	R1	R2	R3			
54A	NH_2_	H	H	0.285	0.467	-0.182
55A	H	CO	H	0.437	0.416	0.020
56A	H	H	NH_2_	0.642	0.467	0.174
57A	H	H	OH	0.748	0.709	0.038
58A	H	OCH_3_	H	0.818	0.918	-0.100
59A	H	H	H	1.23	1.389	-0.159
60A	OH	H	H	0.926	0.709	0.216
61A	CO_2_H	H	H	0.91	0.933	-0.023
62B	NO_2_	H	H	1.549	1.389	0.159
63B	CO_2_H	H	H	0.158	0.416	-0.258
64B	H	CO_2_H	H	0.477	0.416	0.060
65B	Cl	H	H	0.217	0.496	-0.279
66B	H	Cl	H	0.484	0.496	-0.012
67B	H	H	Cl	0.448	0.496	-0.048
68B	H	OH	H	0.65	0.709	-0.059
69B	H	H	NH_2_	0.453	0.467	-0.014
70A	H	H	OCH_3_	0.981	0.918	0.062
71A	F	H	H	0.935	0.933	0.001
72C	H	H	OH	0.906	1.045	-0.139
73C	H	CO_2_H	H	0.921	0.752	0.168
74D	H	CO_2_H	H	0.872	0.752	0.119
75D	H	H	OCH_3_	1.262	1.254	0.007
76D	H	OH	H	0.965	1.045	-0.080
77D	H	NH_2_	H	0.84	0.803	0.036
78D	NH_2_	H	CH	0.6	0.496	0.103
79D	H	H	Cl	0.692	0.496	0.095

**Table 3:** Experimental and predicted biological activity of test set of compounds against BchE.

Compd. No.	Substituents			Experimental Activity	GFA Predicted Activity	GFA Residuals Activity
	R1	R2	R3			
60A	OH	H	H	1.176	1.22	-0.044
20B	H	NH_2_	H	0.448	0.469	-0.021
19C	H	H	H	1.813	1.885	-0.072
21C	H	NO_2_	H	1.247	1.114	0.131
72C	H	H	OH	1.752	1.567	0.184
26C	OH	H	H	1.648	1.771	-0.123
35C	H	H	F	1.942	1.901	0.04
37D	H	NO_2_	H	1.181	1.115	0.065
74D	H	CO_2_	H	0.986	1.115	-0.129
76D	H	OH	H	1.531	1.422	0.108
50D	H	NH_2_	H	1.424	1.369	0.054
77D	H	H	NH_2_	1.136	1.088	0.047
43D	H	NO_2_	H	1.139	1.234	-0.095
7B	NO_2_	H	H	0.991	0.923	0.067
62B	H	Cl	H	1.363	1.285	0.077
86C	H	OCH_3_	H	1.77	1.755	0.014
45D	H	CI	H	1.477	1.522	-0.045
39D	H	NO	H	1.26	1.425	-0.165
59A	H	H	NO_2_	0.561	0.636	-0.075
2A	H	OH	H	0.791	0.803	-0.012

**Figure 1:**
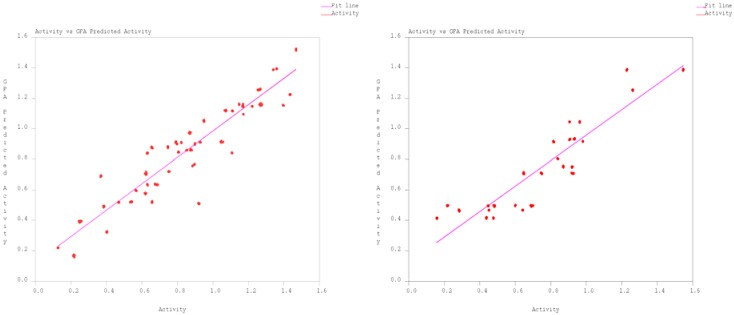
Activity profile against AChE and BChE.

Plots of the experimental and predicted activity for both against AChE and BChE are given in Figure 1.

## 3. Experimental

### 3.1. Data screening and Molecular Modeling

The data used in this study are acetylcholinesterase and butyrylcholinesterase inhibitor activities of a set of 88 *N*-aryl derivatives obtained from the literature [17]. For better scalability, the inhibition constant (Ki)values of the *N*-aryl derivatives were converted into log (Ki) values and this was used as a dependent variable in the study. All the *N*-aryl derivatives were built using INSIGHT-II software and the structures were energy minimized using the cff91 force field. Seventy nine compounds were selected for the QSAR studies in Cerius2 based on the logarithmic value of Ki. Molecular descriptors for each molecule for this QSAR study were calculated in the study table. These descriptors include 2D topological, thermodynamic, structural descriptors and charge dependent descriptors.

### 3.2. Generation of QSAR models using GFA technique

QSAR models were generated using the Genetic Function Approximation (GFA). The GFA technique is a conglomeration of Genetic Algorithm, Friedman’s multivariate adaptive regression splines (MARS) algorithm and Holland’s genetic algorithm to evolve population of equations that best fit the training set data. The GFA algorithm could be a useful technique for searching a large probability space with a large number of descriptors for a small number of molecules. A distinctive feature of GFA is that it produces a population of models (eg.- 100), instead of generating a single model, as do most other statistical methods. The range of variation in this population gives added information on the quality fit and importance of the descriptors. GFA calculations are based on three operators: selection, crossover and mutation. An initial population of equations is generated by random choice of descriptors. The fitness of each equation is scored by Lack- of- Fit (LOF) measure, LOF = LSE / {1- [c + d*p / m]}2 , where LSE is least square error, *c* is the number of basis functions in the model, *d* is the smoothing parameter which controls the number of terms. Crossovers are performed between pairs from the population chosen at random [22] and mutations are performed to add randomness for finding the good GFA equations. In this study, the crossover probability and mutation probability were 0.5, the size of the population 100 and the number of generations was fixed to 100. Several QSAR equations for training sets of 53/61 compounds were obtained using GFA. From those equations, best equations were selected by the statistical measures such as number of compounds in regression, correlation coefficient, square of correlation coefficient r^2^, q^2^, and r^2^_ predictive_ [24]. The correlation coefficient values closer to 1.0 represent the better fit of regression. The squared correlation coefficient (r^2^) is a relative measure of fit by the regression equation. Predictive ability (r^2 ^_pred_) of the selected model was evaluated by test sets of 26/20 compounds for acetylcholinesterase and butyrylcholinesterase, which were not included in training sets.

## 4. Conclusions

Acetylcholinesterase and butyrylcholinesterase are targets for many of the the currently available anti-Alzheimer drugs. In this present work, QSAR analysis on a series of *N*-aryl compounds against both choline esterases was carried out using the GFA technique. The best model was selected based on the statistical parameters. The presented QSAR model has good predictive power (r^2 ^_pred_) and square correlation coefficient (r^2^). The models derived independently for both the targets showed that ALOGP98 (thermodynamic descriptor) and WEINER (charge descriptor) increased the inhibitory potency of N-aryl derivatives against AchE and BChE activity. Thus the descriptors, CHI-V-1-3, CHI-1, WIENER, ALOGP98, KAPPA-1-AM, DIPOLE-MAG, PHI, LogZ and HBOND DONOR seem to determine the activity of the compounds to function as effective AchE and Bche inhibitors. This knowledge can be used for designing more effective chemical entities and may also provide important insights into structural variants leading to the development of novel ChE inhibitors. 
